# Impact of foliar application of iron and zinc fertilizers on grain iron, zinc, and protein contents in bread wheat (*Triticum aestivum* L.)

**DOI:** 10.3389/fnut.2024.1378937

**Published:** 2024-05-14

**Authors:** Sewa Ram, Vipin Kumar Malik, Vikas Gupta, Sneh Narwal, Mohit Sirohi, Ankush, Vanita Pandey, Om Prakash Gupta, Arun Kumar Misra, Gyanendra Singh

**Affiliations:** ^1^ICAR-Indian Institute of Wheat and Barley Research, Karnal, Haryana, India; ^2^ICAR-Indian Agricultural Research Institute, New Delhi, India; ^3^ICAR-National Dairy Research Institute, Karnal, Haryana, India

**Keywords:** agronomic biofortification, grain iron content, grain zinc content, grain protein content, wheat

## Abstract

**Introduction:**

Micronutrient deficiencies, particularly iron (Fe) and zinc (Zn), are prevalent in a large part of the human population across the world, especially in children below 5 years of age and pregnant women in developing countries. Since wheat constitutes a significant proportion of the human diet, improving grain Fe and Zn content in wheat has become important in improving human health.

**Objective:**

This study aimed to quantify the effect of foliar application of iron sulfate heptahydrate (FeSO_4_.7H_2_O) and zinc sulfate heptahydrate (ZnSO_4_.7H_2_O) and their combination on grain Fe and Zn concentrations, as well as grain protein content (GPC). The study also aimed to assess the utility of these applications in large field conditions.

**Methods:**

To address this issue, field experiments were conducted using 10 wheat cultivars and applying a foliar spray of FeSO_4_.7H_2_O (0.25%) and ZnSO_4_.7H_2_O (0.50%) separately (@400 L of solution in water per hectare during each spray) and in combination at two different crop growth stages (flowering and milking) for three consecutive crop seasons (2017–2020). The study used a split-plot design with two replications to assess the impact of foliar application on GFeC, GZnC, and GPC. In addition, an experiment was also conducted to assess the effect of soil (basal) @ 25 kg/ha ZnSO_4_, foliar @ 2 kg/ha, ZnSO_4_.7H_2_O (0.50%), and the combination of basal + foliar application of ZnSO_4_ on the grain micronutrient content of wheat cultivar WB 02 under large field conditions.

**Results:**

GFeC increased by 5.1, 6.1, and 5.9% with foliar applications of FeSO_4_, ZnSO_4_, and their combination, respectively. GZnC increased by 5.2, 39.6, and 43.8% with foliar applications of FeSO_4_, ZnSO_4_, and their combination, respectively. DBW 173 recorded the highest increase in GZnC at 56.9% with the combined foliar application of FeSO_4_ and ZnSO_4_, followed closely by HPBW 01 at 53.0% with the ZnSO_4_ foliar application, compared to the control. The GPC increased by 6.8, 4.9, and 3.3% with foliar applications of FeSO_4_, ZnSO_4_, and their combination, respectively. Large-plot experiments also exhibited a significant positive effect of ZnSO_4_ not only on grain Zn (40.3%, *p* ≤ 0.001) and protein content (*p* ≤ 0.05) but also on grain yield (*p* ≤ 0.05) and hectoliter weight (*p* ≤ 0.01), indicating the suitability of the technology in large field conditions.

**Conclusion:**

Cultivars exhibited a slight increase in GFeC with solitary foliar applications of FeSO_4_, ZnSO_4_, and their combination. In contrast, a significant increase in GZnC was observed with the foliar application of ZnSO_4_ and the combined application of FeSO_4_ and ZnSO_4_. In terms of GPC, the most significant enhancement occurred with the foliar application of FeSO_4_, followed by ZnSO_4_ and their combination. Data demonstrated the significant effect of foliar application of ZnSO_4_ on enhancing GZnC by 39.6%. Large plot experiments also exhibited an increase of 40.3% in GZnC through the foliar application of ZnSO_4_, indicating the effectiveness of the technology to be adopted in the farmer’s field.

## Introduction

Micronutrient deficiency, primarily iron (Fe) and zinc (Zn), is prevalent among the human population, especially in children below the age of 5 years and pregnant women in low- and middle-income countries (1). Approximately two billion people across the world are affected by Fe and Zn deficiencies (2). Fe and Zn deficiencies lead to various health problems such as higher vulnerability to infectious diseases, anemia, disrupted brain function, hampered physical development, and stunting when such micronutrient-deficient diets are consumed over a period of time (3). Since wheat constitutes a significant proportion of the human diet, improving grain Fe and Zn content in wheat has become important in improving human health. Though wheat has large variations among germplasm lines in quantities of protein, carbohydrates, fats, minerals, antioxidants, and vitamins that are required for human health, there is a need to enhance the grain Fe and Zn contents in high-yielding backgrounds. This can be achieved by both genetic manipulations as well as agronomic management of wheat cultivation (4) and delivering wheat rich in Fe and Zn nutrients naturally to everyone, primarily to the segments of populations without access to costly commercially available fortified foods or supplements (5).

Substantial variations in micronutrient concentrations have been reported in wheat grains in different studies (6, 7). Grain Fe content varies typically approximately 1.2-, 1.8-, and 2.9-fold in tetraploid, hexaploid, and diploid wheat cultivars, respectively (8). Diploid progenitors of wheat showed even higher variation, with the highest value of ~110 ppm in some of the diploid accessions (9). The higher content of Fe and Zn in diploids may be due to the presence of very thin grains, higher bran content, and comparatively lesser starchy endosperm content, where micronutrients are concentrated. Recently efforts have been made to improve micronutrient density in commercial wheat cultivars by utilizing diploid progenitors. Commercial wheat cultivars exhibited a lower range in grain Zn concentrations (20–35 mg/kg with an average of 28 mg/kg) in most of the wheat-producing regions (10). Taking these facts into account CIMMYT, Mexico initiated a program for increasing Fe and Zn in the commercial cultivars grown by 12 mg/kg over the global baseline Zn concentration of approximately 25 mg/kg in the target area required for a measurable effect on human health (8). Furthermore, it is also reported in some studies that protein-rich grains have a higher amount of Fe and Zn as compared to low-protein grains (11–13). In addition, soil conditions also cause much more variation than the genotype or species, depending on the nutrient profile of the soil (14). Micronutrient deficiency in grains is caused by some soil factors, i.e., (a) a low/deficient micronutrient availability; (b) pH; (c) a high content/concentration of calcite, bicarbonate ions, and salts; and (d) a high content of available phosphorus and interaction with another nutrient element (14). Multi-location testing of wheat entries under all India-Coordinated Research Projects (AICRPs) on wheat and barley in India demonstrated a large effect of environments on Fe and Zn contents (15). The distinct physiological and biochemical functions of these nutrients in fostering plant growth were examined by Putra et al. (16). Additionally, the pivotal roles of Fe and Zn in the physiology of wheat plants, along with their positive associations with different yield components, have been addressed (17, 18).

Significant improvement in Fe and Zn content by external application of salts containing Fe and Zn has been reported in wheat grains (3, 19–22). As Zn has higher mobility in wheat phloem (23, 24), foliar application of Zn is considered to be the most effective method for improving Zn concentration in wheat grain. Reports indicate that foliar application of Zn salt can increase grain Zn concentration up to 3- or 4-fold, depending on soil status and climate conditions (25–30). Though the level of Fe mobility within the phloem is of intermediate level (31), reports indicated good re-translocation of Fe from the shoot to the grain in wheat (32).

With the prevalence of micronutrient deficiency of these two nutrients, the present study was undertaken to quantify the effect of foliar application of Fe and Zn salts on their concentration in wheat grain using recently released varieties. In addition, large-plot experiments were undertaken to quantify the effect of both foliar application of 0.50% (w/v) of ZnSO_4_·7H_2_O @ 2 kg/ha, soil application of ZnSO_4_ @ 25 kg/ha, and combined application of both foliar spray 0.50% (w/v) of ZnSO_4_·7H_2_O @ 2 kg/ha and basal dose of ZnSO_4_ @ 25 kg/ha on grain Fe and Zn concentration and yield potential to see the utility of foliar application in large field conditions.

## Materials and methods

### The experimental site, plant material, and experimental details

Field experiments were conducted for three consecutive crop seasons 2017–2020 at the Research Farm area of ICAR-Indian Institute of Wheat and Barley Research, Karnal (Haryana), situated at a latitude of 29.6857° N, a longitude of 76.9905° E and an altitude of 240 m above mean sea level (MSL). Ten bread wheat cultivars, namely DBW 173, DBW 88, DBW 90, DPW 621–50, HD 2967, HD 3086, HPBW 01, K 307, WB 02, and WH 1105, were planted in a split-plot design with two replications and three different treatments [foliar application with Fe, Zn, and Fe + Zn] along with control (no foliar application). Treatments included 0.25% (w/v) of an aqueous solution of iron sulfate heptahydrate (FeSO_4_·7H_2_O), 0.50% (w/v) of an aqueous solution of zinc sulfate heptahydrate (ZnSO_4_·7H_2_O), and a combination of FeSO_4_·7H_2_O [0.25% (w/v)] + ZnSO_4_·7H_2_O [0.50% (w/v)] following the methods described by Aciksoz et al. (33) and Cakmak et al. (34). Two foliar sprays of all three treatments were applied @400 L of solution per hectare at two different crop growth stages (flowering and milking) during evening hours. Thus, the total amount of FeSO_4_·7H_2_O and ZnSO_4_·7H_2_O used was 2.0 kg and 4.0 kg, respectively for both sprays. In addition, a large-scale experiment (1 acre of land) was conducted at ICAR-NDRI, Karnal (Haryana) during 2019–2020 using the cultivar WB 02 for the assessment of the effect of the foliar application of aqueous solution (0.50% w/v) of ZnSO_4_·7H_2_O @ 2 kg/ha, basal application of ZnSO_4_ @ 25 kg/ha, and combined application of both foliar spray 0.50% (w/v) of ZnSO_4_·7H_2_O @ 2 kg/ha and basal dose of ZnSO_4_ @ 25 kg/ha at two different crop growth stages (flowering and milking) on GFeC, GZnC, and GPC along with the grain yield.

Every year, sowing was done in the second week of November in a well-prepared field at 25 cm row spacing with a plot size of 3 rows of 2 m. Recommended doses of fertilizer (120 kg N, 60 kg P_2_O_5_, and 40 kg K_2_O per ha) were used with full doses of K_2_O and P_2_O_5_ applied at sowing; nitrogen was applied in three split doses: 60 kg N per ha as basal dose at the time of sowing, 30 kg N per ha at first irrigation (21 days after sowing), and 30 kg N per ha at second irrigation (45 days after sowing). Finally, trials were harvested in the third week of April of the subsequent years at maturity. All the recommended packages of practices were followed for raising a good crop.

### Data collection and statistical analysis

Twenty-five to 30 spikes from each replication of treatment were bulked separately and hand-threshed in a clean cloth bag by beating with a wooden stick, and the grains were separated from the husk in a plastic tray. After threshing, 15–20 g seed from each replication was used for measuring grain iron concentration (GFeC), grain zinc concentration (GZnC), and grain protein content (GPC). GFeC and GZnC were measured using energy dispersive X-ray fluorescence (ED-XRF) model X-Supreme 8,000 (M/s Oxford Inc., USA). Whereas, GPC was estimated using Foss Infratec TM 1241 Grain Analyzer, and the final value of GPC was calculated on a 12% moisture basis. Finally, data for GFeC, GZnC, and GPC were analyzed for descriptive statistics, analysis of variance (ANOVA), and Pearson’s correlation coefficient using the publicly available statistical analysis platform R version 4.2.1 for Windows (R core team), and the % change over control was calculated and their graphical representation was presented using MS excel.

## Results

**Figure 1:**
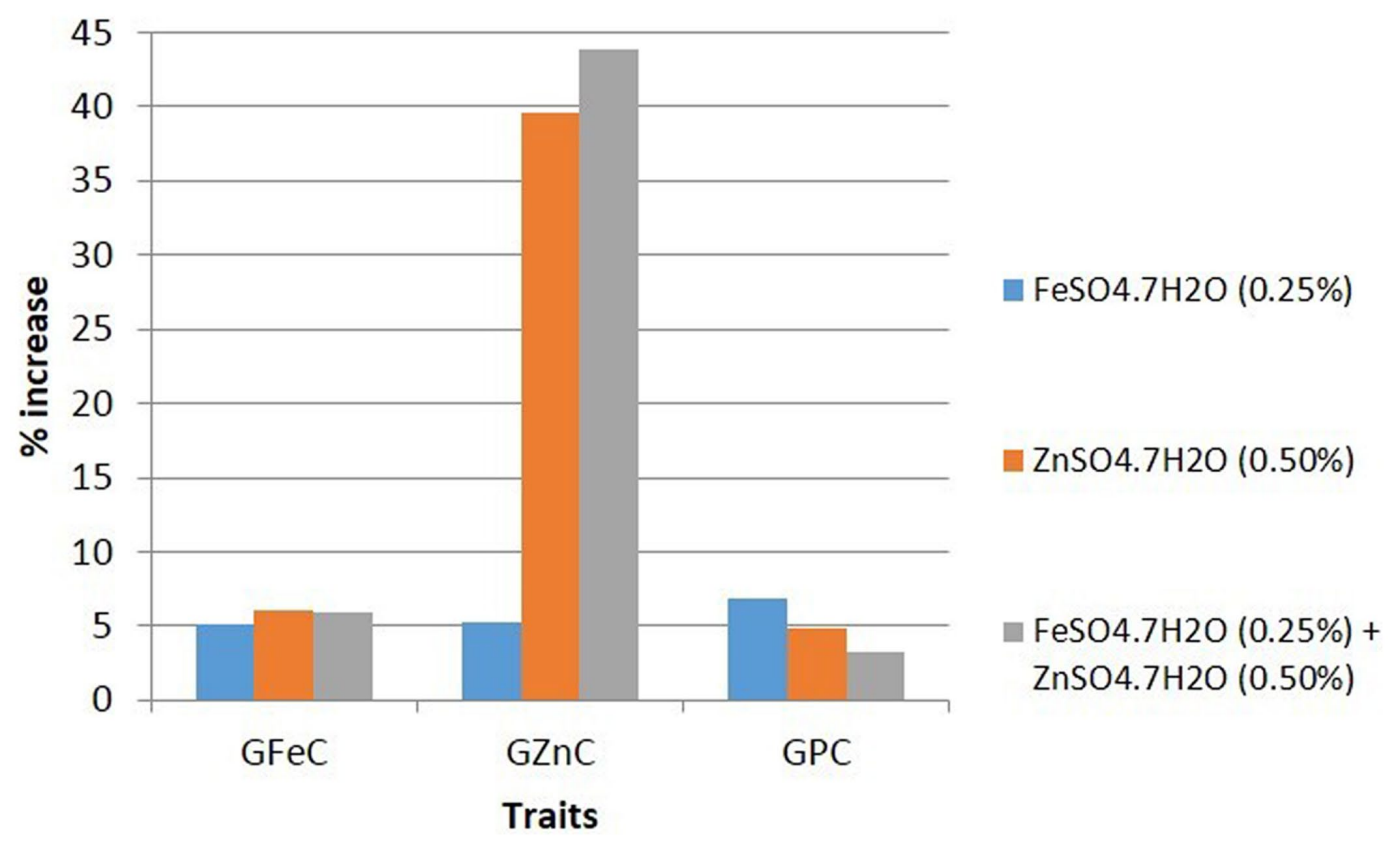
Overall effect of foliar application of FeSO_4_, ZnSO_4_, and their combination on GFeC, GZnC, and GPC over control of 10 wheat cultivars grown at Karnal during 2017–2020.

Data on grain Fe, Zn, and protein contents were analyzed for summary statistics, in which GFeC ranged from 30.6 to 45.3 mg/kg (mean = 38.09 mg/kg and CV = 9.1%), GZnC varied from 21.0 to 36.3 mg/kg (mean = 27.32 mg/kg and CV = 11.3%), and GPC ranged from 8.4 to 12.8% (mean = 10.71% and CV = 8.9%) under control conditions. While GFeC, GZnC, and GPC varied from 32.9 to 48.8 mg/kg (mean = 40.41 mg/kg and CV = 10.3%), 22.3 to 37.4 mg/kg (mean = 28.75 mg/kg and CV = 12.7%), and 10.0 to 12.8% (mean = 11.41% and CV = 5.8%), respectively, foliar application of FeSO_4_ increased GFeC, GZnC, and GPC by 5.1, 5.2, and 6.8%, respectively, as compared to the control. However, GFeC, GZnC, and GPC ranged from 33.6 to 49.0 mg/kg (mean = 40.38 mg/kg and CV = 9.7%), 29.8 to 49.2 mg/kg (mean = 37.75 mg/kg and CV = 12.8%), and 9.8 to 12.7% (mean = 11.20% and CV = 5.4%), respectively, by the foliar application of ZnSO_4_, increasing GFeC, GZnC, and GPC by 6.1, 39.6, and 4.9%, respectively. Interestingly, a significant and large increase in GZnC (39.6%) was observed by the foliar application of ZnSO_4_. Similarly, GFeC, GZnC, and GPC ranged from 32.4 to 52.0 mg/kg (mean = 40.38 mg/kg and CV = 12.7%), 27.8 to 52.5 mg/kg (mean = 38.94 mg/kg and CV = 13.8%), and 9.3 to 12.2% (mean = 11.04% and CV = 6.1%), respectively, by the combined foliar application of FeSO_4_ and ZnSO_4_, increasing GFeC, GZnC, and GPC by 5.9, 43.8, and 3.3%, respectively (Table 1 and Figure 1).

**Table 1:** Overall performance of the entire set of wheat cultivars for GFeC, GZnC, and GPC using the foliar application of FeSO_4_, ZnSO_4_, and their combination along with control grown at Karnal during 2017–2020.

Treatments	Traits	Min	Max	Mean ± SEM	Range	Variance	StdDev	CV (%)	% increase
Control	GFeC (mg/kg)	30.6	45.3	38.09 ± 0.45	14.7	12.1	3.5	9.1	–
	GZnC (mg/kg)	21.0	36.3	27.32 ± 0.40	15.3	9.5	3.1	11.3	–
	GPC (%)	8.4	12.8	10.71 ± 0.12	4.4	0.9	1.0	8.9	–
+ FeSO_4_	GFeC (mg/kg)	32.9	48.8	40.04 ± 0.53	15.9	17.1	4.1	10.3	5.1
	GZnC (mg/kg)	22.3	37.4	28.75 ± 0.47	15.1	13.4	3.7	12.7	5.2
	GPC (%)	10.0	12.8	11.41 ± 0.07	2.8	0.3	0.6	5.0	6.8
+ ZnSO_4_	GFeC (mg/kg)	33.6	49.0	40.38 ± 0.51	15.4	15.4	3.9	9.7	6.1
	GZnC (mg/kg)	29.8	49.2	37.75 ± 0.62	19.4	23.4	4.8	12.8	39.6
	GPC (%)	9.8	12.7	11.20 ± 0.08	2.9	0.4	0.6	5.4	4.9
+ (FeSO_4_ + ZnSO_4_)	GFeC (mg/kg)	32.4	52.0	40.35 ± 0.66	19.6	26.4	5.1	12.7	5.9
	GZnC (mg/kg)	27.8	52.5	38.94 ± 0.69	24.7	28.8	5.4	13.8	43.8
	GPC (%)	9.3	12.2	11.04 ± 0.09	2.9	0.5	0.7	6.1	3.3

Min, minimum; Max, maximum; SEM, standard error of a mean; StdDev, standard deviation; CV, coefficient of variation; GFeC, grain iron concentration; GZnC; grain zinc concentration; GPC, grain protein content.

ANOVA showed that the genotypic effect was significant for GZnC (*p* ≤ 0.01) and GPC (*p* ≤ 0.05) and no effect for GFeC. Though the environment had a significant effect on GFeC and GZnC, the interactive effect of genotype and year was significant only on GZnC (*p* ≤ 0.05). On the other hand, treatments and their interaction with environments showed significant effects (*p* ≤ 0.01) for all three traits (Table 2). There was a significant positive correlation between GFeC and GZnC (*r* = 0.60; *p* ≤ 0.01) under control conditions, as well as the foliar application of FeSO_4_ (*r* = 0.70 and *p* ≤ 0.01), ZnSO_4_ (*r* = 0.25; *p* ≤ 0.05), and the combined application of FeSO_4_ and ZnSO_4_ (*r* = 0.26; *p* ≤ 0.05). There was also a significant positive correlation between GZnC and GPC (*r* = 0.28; *p* ≤ 0.05) using the combined foliar application of FeSO_4_ and ZnSO_4_ (Table 3).

**Table 2:** Analysis of variance (ANOVA) of 10 wheat cultivars for GFeC, GZnC, and GPC grown at Karnal during 2017–2020.

Source	d.f	GFeC (mg/kg)	GZnC (mg/kg)	GPC (%)
Year	2	1170.78**	191.83*	0.90
Replication	3	19.22	15.59	6.02**
Cultivars	9	20.24	77.11**	1.30*
Year: cultivars	18	15.40	24.81*	0.73
Pooled error(a)	27	10.95	11.94	0.46
Treatment	3	71.96**	2162.61**	5.34**
Cultivars: treatment	27	4.87	12.70*	0.17
Year: treatment	6	24.22**	183.88**	1.36**
Year: cultivars: Treatment	54	2.89	8.15	0.25
Pooled error(b)	90	6.67	7.18	0.41
Total	239			

d.f, degree of freedom; GFeC, grain iron concentration; GZnC; grain zinc concentration; GPC, grain protein content; *, significant at a *p*-value of ≤0.05; **, significant at a *p*-value of ≤0.01.

**Table 3:** Pearson’s correlation coefficient of the entire set of wheat cultivars for GFeC, GZnC, and GPC using the foliar application of FeSO_4_, ZnSO_4_, and their combination along with control grown at Karnal during 2017–2020.

Treatments	Traits	GFeC (mg/kg)	GZnC (mg/kg)	GPC (%)
Control	GFeC (mg/kg)	1	0.60**	0.21
	GZnC (mg/kg)		1	−0.22
	GPC (%)			1
+ FeSO_4_	GFeC (mg/kg)	1	0.70**	0.09
	GZnC (mg/kg)		1	−0.19
	GPC (%)			1
+ ZnSO_4_	GFeC (mg/kg)	1	0.26*	−0.10
	GZnC (mg/kg)		1	−0.002
	GPC (%)			1
+ (FeSO_4_ + ZnSO_4_.7H_2_O)	GFeC (mg/kg)	1	0.25*	0.17
	GZnC (mg/kg)		1	0.28*
	GPC (%)			1
Pooled analysis	GFeC (mg/kg)	1	0.37**	0.16*
	GZnC (mg/kg)		1	0.05
	GPC (%)			1

GFeC, grain iron concentration; GZnC, grain zinc concentration; GPC, grain protein content; *, significant at a *p*-value of ≤0.05; **, significant at a *p*-value of ≤0.01.

The mean performance of wheat cultivars based on the LSD test exhibited significant differences for GZnC only with the foliar application of ZnSO_4_ and the combined foliar application of FeSO_4_ and ZnSO_4_. The cultivar WB 02 accumulated the highest GZnC (42.7 mg/kg), followed by wheat cultivars HPBW 01, WH 1105, K 307, HD 3086, DBW 173, HD 2967, DBW 90, DPW 621–50, and DBW 88 using the foliar application of ZnSO_4_. Whereas, cultivar WB 02 accumulated the highest GZnC (44.4 mg/kg), followed by wheat cultivars K 307, DBW 173, WH 1105, HPBW 01, DPW 621–50, HD 3086, DBW 90, DBW 88, and HD 2967 using the combined foliar application of FeSO_4_ and ZnSO_4_. However, there was no significant varietal difference in mean GFeC and GPC between the foliar application of either FeSO_4_ or ZnSO_4_ or the combined foliar application of FeSO_4_ and ZnSO_4_ (Supplementary Table S1).

**Figure 2:**
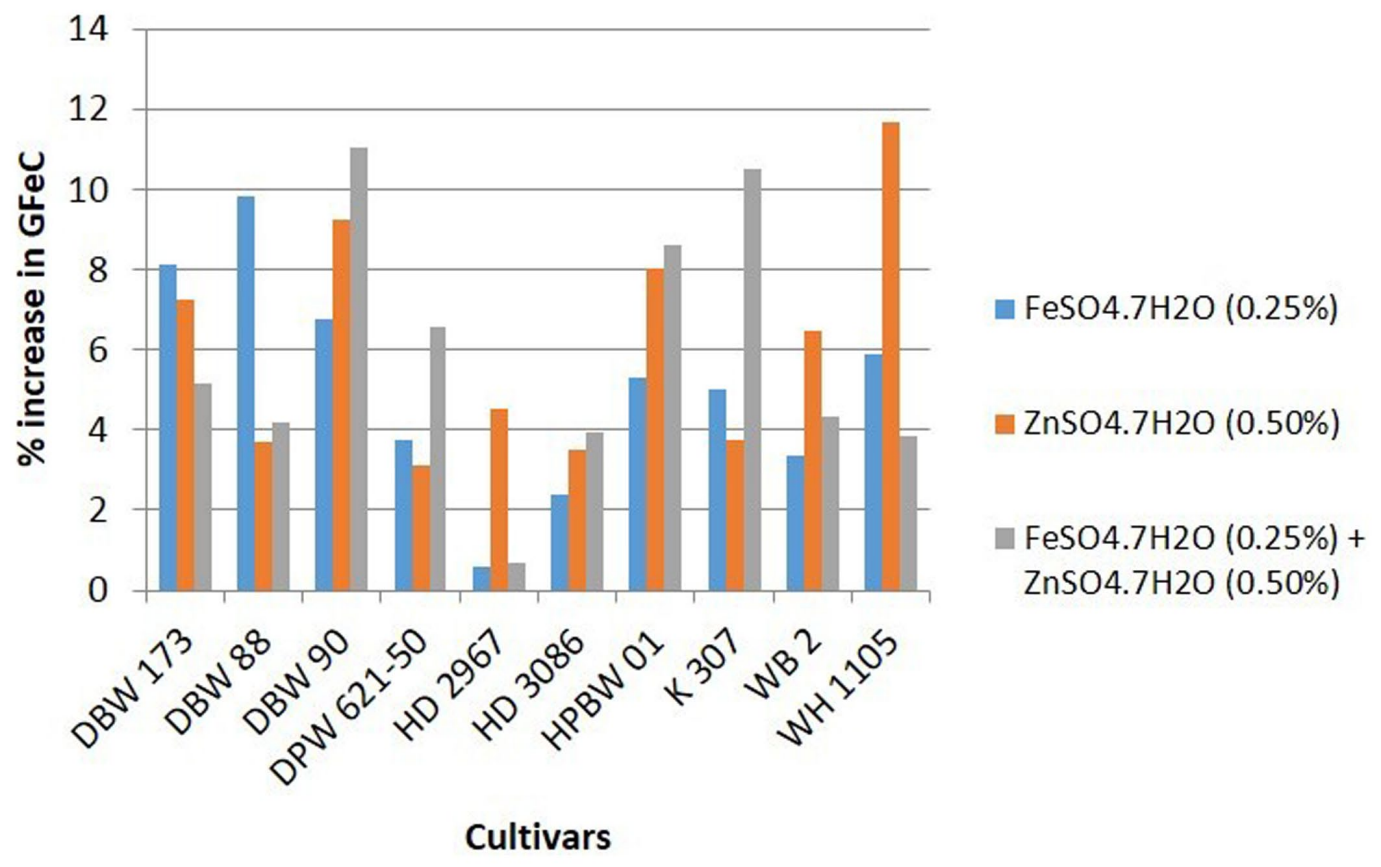
Variety-wise percent change in GFeC over control by the foliar application of FeSO_4_, ZnSO_4_, and their combination (wheat cultivars grown at Karnal during 2017–2020).

**Figure 3:**
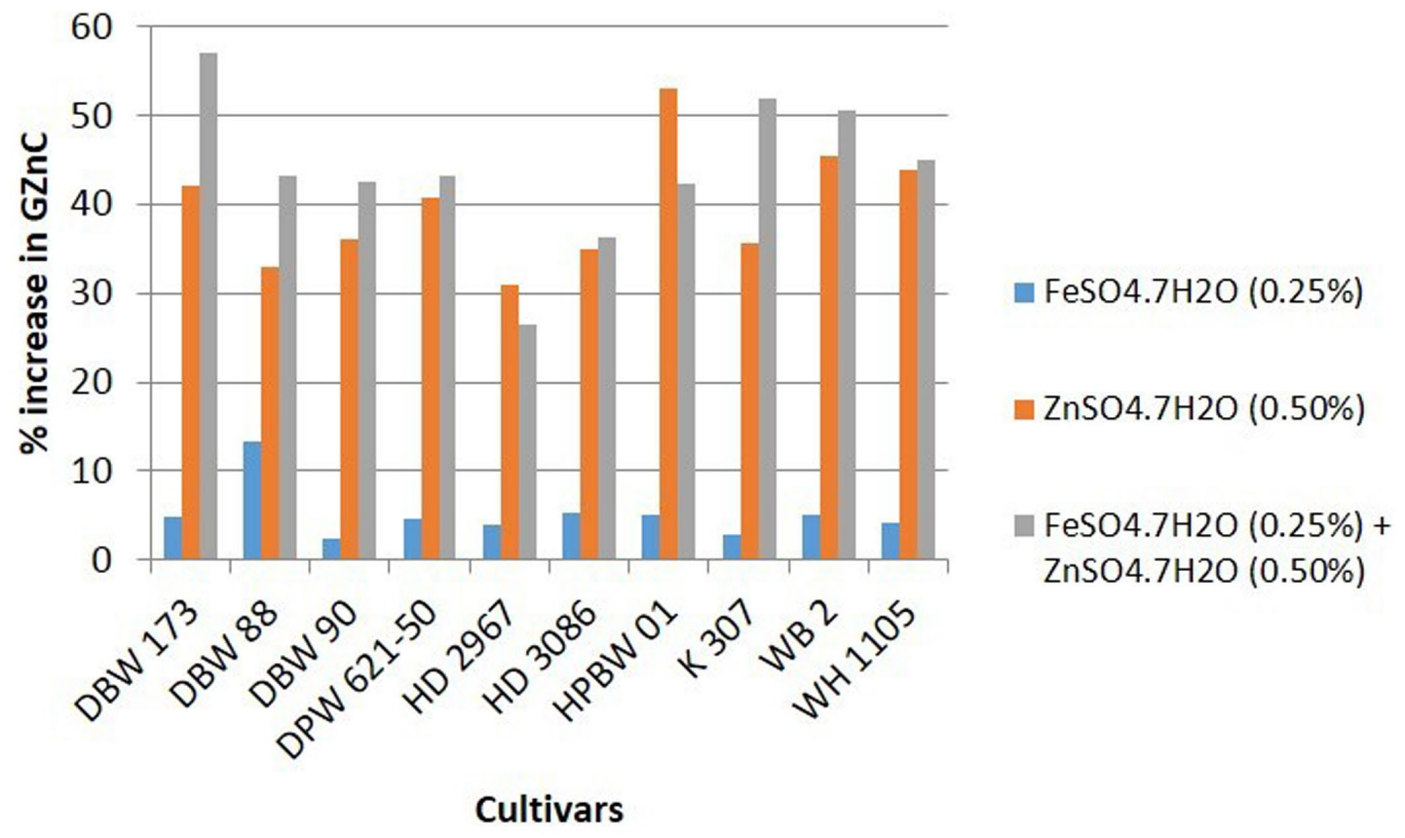
Variety-wise percent change in GZnC over control by the foliar application of FeSO_4_, ZnSO_4_, and their combination (wheat cultivars grown at Karnal during 2017–2020).

**Figure 4:**
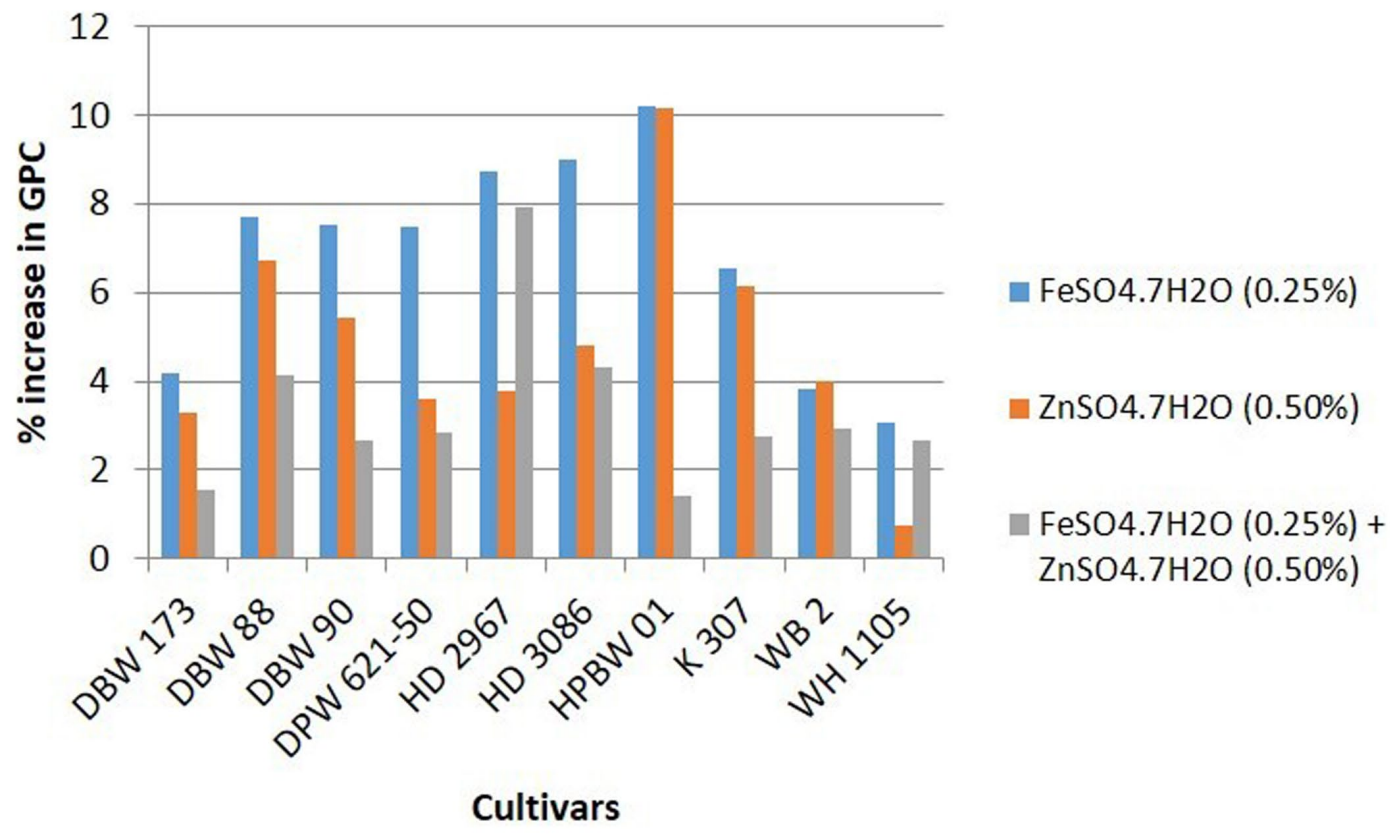
Variety-wise percent change in GPC over control by the foliar application of FeSO_4_, ZnSO_4_, and their combination (wheat cultivars grown at Karnal during 2017–2020).

Details of variety-wise changes in GFeC, GZnC, and GPC in different experiments are given in Supplementary Table S2 and Figures 2–4. As compared to control conditions, the highest increase in GFeC was observed in the cultivar WH 1105 (11.6% higher) by foliar application of ZnSO_4_ (Supplementary Table S2 and Figure 2); the highest increase in GZnC was observed in DBW 173 (56.9%) by the combined foliar application of FeSO_4_ and ZnSO_4_ (Supplementary Table S2 and Figure 3); and the highest increase in GPC (10.2%) was exhibited in HPBW 01 by foliar applications of both FeSO_4_ and ZnSO_4_ salts independently (Supplementary Table S2 and Figure 4).

**Figure 5:**
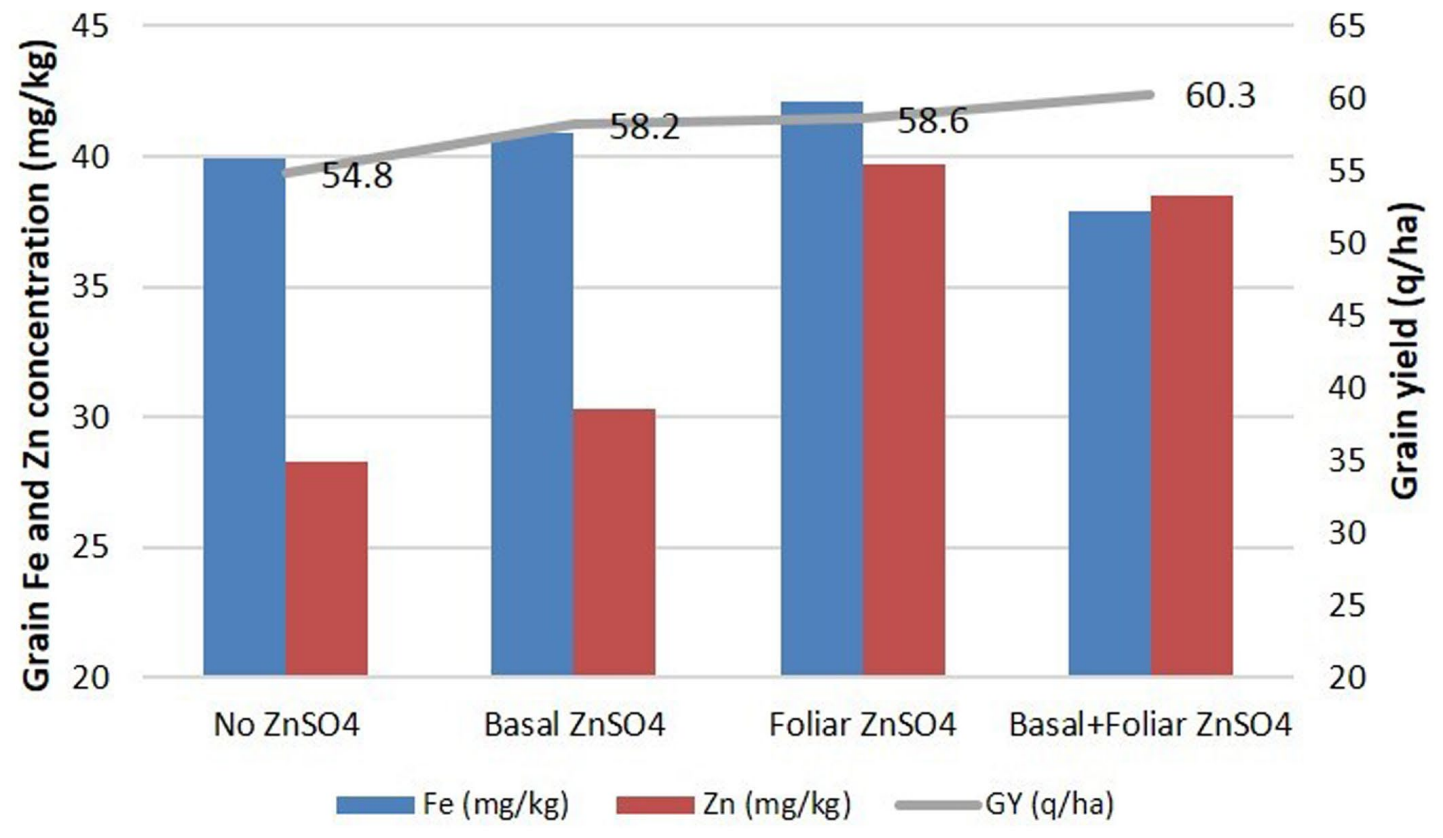
Effect of Zn treatments (no Zn, basal ZnSO_4_, foliar ZnSO_4_, and basal + foliar ZnSO_4_) on GFeC, GZnC, and grain yield of cultivar WB 02 in an experiment conducted on 1 acre of land at Karnal during 2019–2020.

With the high significant effect of ZnSO_4_ on Zn content, an experiment was conducted in an area of 1 acre to assess the impact of the application in large field conditions. The foliar spray and combination of basal + foliar application of ZnSO_4_ exhibited a very large increase (40.3%) (*p* ≤ 0.001) in the Zn content, while basal application showed a comparatively smaller increase (7.0%). GZnC was 28.3 mg/kg, 30.3 mg/kg, 39.7 mg/kg, and 39.7 mg/kg, while GFeC was 39.9 mg/kg, 40.9 mg/kg, 42.1 mg/kg, and 37.9 mg/kg under treatments such as no Zn, basal ZnSO_4_, foliar ZnSO_4_, and basal+foliar ZnSO_4_, respectively. Grain yield also increased significantly, showing 54.8 q/ha, 58.2 q/ha, 58.6 q/ha, and 60.3 q/ha under treatments such as no Zn, basal ZnSO_4_, foliar ZnSO_4_, and basal+foliar ZnSO_4_, respectively. In addition to GZnC, significant increases were observed for GPC (*p* ≤ 0.05) and hectoliter weight (*p* ≤ 0.01), by the application of foliar ZnSO4 spray (Figure 5).

## Discussion

Fe and Zn are essential nutrients for the growth and development of both plants and animals. At present, an increase in crop-nutritional value is of utmost importance because a large part of the human population is suffering from micronutrient deficiency. A sustainable and cost-efficient approach being used for the reduction of mineral malnutrition in developing countries is agronomic biofortification, among all current strategies. Therefore, this study was conducted to address the effectiveness of the foliar application of two different micronutrient concentrations of Fe and Zn salts (alone and in combination) on three important nutritional quality traits of wheat, i.e., GFeC, GZnC, and GPC. ANOVA results indicated significant differences among genotypes (for GZnC and GPC except for GFeC), treatments (for GFeC, GZnC, and GPC), years [(for GFeC, GZnC except for GPC), and the year × treatment interaction (for GFeC, GZnC, and GPC)], suggesting the influence of environment on the expression of these traits. Other studies also showed that the three traits are highly influenced by the genotype × environment interaction and are controlled by polygenes (35). The notable genotype by environment (G × E) interaction observed in our study may arise from differences in micronutrient levels within the soil, as previously reported (36) with genotype as the primary determinant of variation in GFeC, GZnC, and GPC (37–40).

### Impact of foliar application of FeSO_4_ on GFeC and GZnC

Over the years, GFeC and GZnC ranged from 30.6 to 45.3 mg/kg with an average value of 38.1 mg/kg and 21.0 to 36.3 mg/kg with an average value of 27.3 mg/kg, respectively, under control conditions. Various studies also reported diverse GFeC and GZnC levels in wheat: 28.5–46.3 mg/kg and 33.6–65.6 mg/kg (41); 17.8–49.7 mg/kg and 24.5–44.3 mg/kg (42); and 24.2–48.5 mg/kg and 19.4–47.7 mg/kg (43). Previous studies reported an average GFeC of 35.0 mg/kg at CIMMYT, Mexico (44) and an average GZnC of 27.3 mg/kg in 160 Chinese ancient wheat cultivars (45). In this experiment, foliar application of FeSO_4_ marginally increased average GFeC by 5.1% over the control. Prior reports also indicated no significant influence of foliar application of Fe fertilizers, either in inorganic or chelated form, on grain Fe concentration in Canadian wheat cultivars (46). The grain Fe concentration increased to approximately 21% in Iran (47), 28% in China, 14% with FeEDTA, and 10% with FeSO_4_ in central Anatolia, Turkey, with no significant effect on Zn content (33). The lesser increase in Fe content in wheat grains following foliar Fe application may be due to limited penetration into leaf tissues and restricted phloem mobility (48, 49). Additionally, the effectiveness of different forms of Fe salts, including FeSO_4_, FeEDTA, FeDTPA, FeEDDHA, and Fe-citrate in addressing Fe deficiency varies significantly based on factors such as solubility, stability, leaf cuticle penetration, mobility, and translocation within leaf tissues (50, 51). Though negative effects of foliar sprays of different doses of Fe salts on grain Zn concentration have been reported, leading to lower Zn content as compared to the control (52), in this investigation, GZnC increased by 5.2% with the sole application of Fe salt to the leaves. The negative effect of Fe application on Zn content has been attributed to the antagonistic relationship between Fe and Zn elements (53). Similar findings regarding the interaction of Fe with Zn and other elements were observed in, paddy (54), and rice (55), with a decline in Zn translocation as Fe levels increased. Our findings on the foliar application of FeSO_4_ are consistent with the findings of Pahlavan-Rad and Pessarakli (47), who concluded a 21% increase in grain Fe and a 13% increase in grain Zn concentration in wheat.

### Impact of foliar application of ZnSO_4_ on GFeC and GZnC

It is interesting to note that foliar Zn salt application improved GFeC by 6.8%, from 38.1 to 40.4 mg/kg, as compared to the controlled condition. Some of the previous studies also found that foliar Zn salt application significantly improved GFeC of the bran and embryo parts of the wheat grain, which may be due to the ability of Zn-binding compounds to act as sinks for Fe transport and storage in grains (56, 57). This increase in GFeC following foliar treatment of Zn salt was also seen in potatoes and had no antagonistic impact on the tuber Fe content (58). Our findings from the foliar application of ZnSO4, in collaboration with Pahlavan-Rad and Pessarakli (47) resulted in a 99% boost in Zn concentration and an 8% increase in Fe concentration in wheat grains. Foliar spray of ZnSO_4_ increased Zn content significantly (*p* ≤ 0.001) by 39.6% compared to controlled conditions, from 27.3 to 37.8 mg/kg (Table 1). This significant increase in Zn content in wheat grains may be attributed to the improved mobility of Zn in the phloem and its efficient translocation into developing wheat grains (24, 59). Not only does foliar Zn salt application alone result in high grain Zn concentration, but also the combined application of Fe and Zn boosts Zn concentration in wheat grains in a similar way. Several other reports also showed enhanced wheat grain Zn content by 58% (60) and 83% (22) with foliar application of Zn salt. In addition, other studies reported increased grain Zn concentrations by 27% in rice and 9% in maize (61) with the foliar application of Zn salt. To further substantiate the significant effect of ZnSO_4_ spray on Zn content applicable to large field conditions, an experiment was conducted in an area of 1 acre using both foliar and basal application. There was a 7% increase in Zn content by the basal application of ZnSO_4_, while it was 40.3% by foliar application. This demonstrated that the foliar application of ZnSO_4_ can be used to enhance Zn content significantly in farmer’s field conditions.

### Impact of combined foliar application of FeSO_4_ and ZnSO_4_ on GFeC and GZnC

Combined application of FeSO_4_ and ZnSO_4_ led to a 5.9% increase in GFeC as compared to control conditions. It ranged from 32.4 to 52.0 mg/kg, with an average of 40.35 mg/kg. It is worth noting that a lesser increase in GFeC through agronomic biofortification occurred as compared to GZnC. This may likely be due to the limited phloem mobility of Fe from leaves to grains (62). These results corroborate earlier findings (63, 64), indicating a weak association between GFeC and Fe salts when applied via foliar or soil route. However, in one of the studies, an increase in GFeC up to 28% was observed following foliar Fe salt application (65). Promisingly, the combined application of Fe and Zn salts increased GZnC by 43.8% over control and ranged from 27.8 to 52.5 mg/kg, with an average of 38.9 mg/kg. The increase in GZnC was similar to the foliar application of Zn salt alone. Similarly, several other reports also showed a higher accumulation of Zn content by foliar application (22, 60). This may be due to the better mobility of the Zn-binding compound and the ready translocation of the Zn element in the phloem to emerging wheat grains (53, 56, 59).

### Impact of foliar application of FeSO_4_, ZnSO_4_, and their combination on GPC

The foliar application of Fe, Zn, and a combination of Fe and Zn salts increased GPC by 6.8, 4.9, and 3.3%, respectively, indicating a higher influence of the application of FeSO_4_ on the GPC. GPC ranged from 10.0 to 12.8%, averaging 11.4% with Fe salt application, 9.8 to 12.7%, averaging 11.2% with Zn salt application, and 9.3 to 12.2%, averaging 11.0% with combined Fe and Zn salt application. Combined application of Fe and Zn led to a lower increase in protein content, which may be because of some antagonistic effect of the Fe and Zn combination. Several other reports also showed improvements in GPC by the application of Fe and Zn salts in wheat (11, 60, 66, 67). Previous studies have indicated that foliar application of micronutrients such as FeSO_4_ and ZnSO_4_ enhanced grain weight as well as grain and straw yield (26, 30, 68–70). Significant positive correlation among GFeC, GZnC, and GPC provided additional support that the application of Fe and Zn salts can enhance protein content. Earlier reports (40, 71–73) similarly found a positive correlation between Fe and Zn content and the GPC in wheat. They suggested that the accumulation of Fe and Zn in wheat grains is influenced by grain protein levels.

### Varietal response to the foliar application of FeSO_4_, ZnSO_4_, and their combination

Varietal responses to various foliar sprays of micronutrients displayed significant differences (Supplementary Table S1). The average performance of wheat cultivars, as determined by LSD testing, revealed significant differences only with the application of ZnSO_4_ and a combination of FeSO_4_ and ZnSO_4_ via foliar spray, specifically in the case of GZnC. Varietal responses varied in terms of absorption, accumulation, and translocation, factors primarily determined by inherent genetic potential. Notably, in our study, WB 02, bred with a focus on biofortification traits, exhibited a heightened response in GZnC to both foliar and basal application of mineral fertilizers. It is recommended to assess genotype performance for agronomic fortification through micronutrient application to attain accurate outcomes. Identifying and endorsing lines with superior grain micronutrient content for cultivation are imperative in combating malnutrition.

### Effect of foliar, soil, and their combined application of ZnSO_4_ using large plots on GFeC, GZnC, GPC, grain yield, and hectoliter weight

An experiment of basal (soil) application and foliar spray of ZnSO_4_ was conducted using 1 acre of land to evaluate the suitability of the technology in farmer’s field conditions. The data exhibited that the foliar application of ZnSO_4_ significantly increased GZnC (*p* ≤ 0.001), GPC (*p* ≤ 0.05), grain yield (*p* ≤ 0.05), and hectoliter weight (*p* ≤ 0.01) but had no effect on GFeC. So, a large-scale experiment further demonstrated the positive effect of ZnSO_4_ on GPC and hectoliter weight as well as on yield. An additive effect on Zn and protein concentrations was previously reported in wheat grain (74). Zn and Fe salts applied via soil and foliar methods, along with 120 kg N/ha at sowing, led to a 46% increase in Zn and a 35% increase in Fe concentration (75). A positive relationship between Zn and N is observed with increased protein content (76). In addition, reports also showed synergistic interaction among the plant nutrients that led to enhanced crop growth and grain yield (77, 78). Studies have shown that grain yield can lead to a “dilution effect” on grain Zn concentration across various cultivars or fields (79, 80). However, in this study, a dilution effect on grain yield was also observed in the basal + foliar spray treatment, although it was not significant. Therefore, the foliar application of ZnSO_4_ can be used as a technology for increasing the yield and quality of wheat in large-field conditions.

## Conclusion

The present investigation was undertaken to quantify the effect of foliar application of Fe and Zn salts on Fe, Zn, and protein concentrations in wheat grain. The data demonstrated the significant effect of foliar application of ZnSO_4_ on grain Zn and protein contents. The large-plot experiments also exhibited the significant positive effect of ZnSO_4_ not only on grain Zn and protein content but also on grain yield and hectoliter weight, indicating the suitability of the technology in the farmer’s field. The cultivars exhibited a slight increase in GFeC with solitary foliar applications of FeSO_4_, ZnSO_4_, and their combination. In contrast, a significant increase in GZnC was observed with the foliar application of ZnSO_4_ and the combined application of FeSO_4_ and ZnSO_4_. In terms of GPC, the most significant enhancement occurred with the foliar application of FeSO_4_, followed by ZnSO_4_ and their combination. The shortcoming of the implementation of the foliar application adds to the overall cultivation expenses for farmers. The efficacy of foliar spraying of micronutrients is heavily influenced by environmental variables such as the timing of application, temperature, wind speed, and rainfall, which could potentially hinder their effectiveness. However, keeping in view the potential of this technology to combat micronutrient malnutrition, it is better to adopt agronomic biofortification through foliar application.

## Data availability statement

The original contributions presented in the study are included in the article/Supplementary material, further inquiries can be directed to the corresponding author.

## Author contributions

SR: Conceptualization, Formal analysis, Funding acquisition, Investigation, Methodology, Supervision, Validation, Visualization, Writing – original draft, Writing – review & editing, Data curation, Project administration, Resources. VKM: Data curation, Formal analysis, Software, Visualization, Writing – original draft, Writing – review & editing. VG: Conceptualization, Formal analysis, Methodology, Visualization, Writing – review & editing. SN: Conceptualization, Methodology, Supervision, Visualization, Writing – review & editing, Data curation, Project administration. MS: Data curation, Formal analysis, Visualization, Writing – original draft. Ankush: Data curation, Writing – original draft. VP: Visualization, Writing – review & editing, Conceptualization, Data curation, Investigation, Validation. OPG: Visualization, Writing – review & editing, Data curation, Investigation, Validation. AM: Data curation, Investigation, Methodology, Supervision, Validation, Visualization, Writing – review & editing. GS: Investigation, Project administration, Supervision, Visualization, Writing – review & editing.
