# Supplementary material for: Impact of foliar application of iron and zinc fertilizers on grain iron, zinc, and protein contents in bread wheat (Triticum aestivum L.)
Source: Front Nutr. 2024 May 14;11:1378937. doi: 10.3389/fnut.2024.1378937 (PMC11130500; doi:10.3389/fnut.2024.1378937)
Supplement: Supplementary file 1 [file Table_1.doc]

**Supplementary table 1** Performance of ten wheat cultivars using three different foliar applications along with control on GFeC, GZnC and GPC grown at Karnal during 2017-20

| Cultivars | GFeC (mg/kg) | | | | GZnC (mg/kg) | | | | GPC (%) | | | |
| --- | --- | --- | --- | --- | --- | --- | --- | --- | --- | --- | --- | --- |
|  | Control | + Fe | + Zn | + (Fe+Zn) | Control | + Fe | + Zn | + (Fe+Zn) | Control | + Fe | + Zn | + (Fe+Zn) |
| DBW 173 | 38.2 | 41.1 | 40.8 | 40.2 | 26.0^a^ | 27.3^a^ | 36.6^bc^ | 40.5^abc^ | 11.0 | 11.5 | 11.4 | 11.2 |
| DBW 88 | 36.7 | 40.4 | 38.1 | 38.3 | 26.1^a^ | 29.7^a^ | 34.7^c^ | 37.4^bc^ | 10.7 | 11.5 | 11.4 | 11.1 |
| DBW 90 | 37.3 | 39.9 | 40.8 | 41.5 | 26.3^a^ | 27.0^a^ | 35.7^c^ | 37.5^bc^ | 10.3 | 11.0 | 10.8 | 10.6 |
| DPW 621-50 | 38.3 | 39.9 | 39.6 | 41.0 | 25.4^a^ | 26.6^a^ | 35.5^c^ | 36.2^c^ | 11.0 | 11.8 | 11.5 | 11.2 |
| HD 2967 | 38.7 | 38.9 | 40.2 | 38.9 | 28.2^a^ | 29.3^a^ | 36.4^c^ | 35.3^c^ | 10.6 | 11.5 | 11.0 | 11.4 |
| HD 3086 | 39.8 | 40.7 | 41.2 | 41.4 | 27.9^a^ | 29.5^a^ | 37.3^abc^ | 37.5^bc^ | 10.4 | 11.3 | 10.9 | 10.8 |
| HPBW 01 | 37.8 | 39.9 | 40.7 | 41.0 | 27.6^a^ | 29.0^a^ | 41.9^ab^ | 39.0^bc^ | 10.4 | 11.3 | 11.4 | 10.5 |
| K 307 | 39.3 | 41.3 | 40.7 | 43.5 | 28.1^a^ | 28.9^a^ | 37.5^abc^ | 42.2^ab^ | 10.7 | 11.4 | 11.4 | 11.1 |
| WB 2 | 38.5 | 39.9 | 41.0 | 40.2 | 30.1^a^ | 31.6^a^ | 42.7^a^ | 44.4^a^ | 10.9 | 11.3 | 11.2 | 11.1 |
| WH 1105 | 36.4 | 38.6 | 40.7 | 37.5 | 27.5^a^ | 28.8^a^ | 39.2^abc^ | 39.4^abc^ | 11.2 | 11.5 | 11.3 | 11.5 |
| Average | 38.1 | 40.1 | 40.4 | 40.4 | 27.3 | 28.8 | 37.8 | 38.9 | 10.7 | 11.4 | 11.2 | 11.1 |

GFeC, Grain iron concentration; GZnC, Grain zinc concentration; GPC, Grain protein content; + Fe, Foliar application of FeSO_4_.7H_2_O (0.25 %); +Zn, Foliar application of ZnSO_4_.7H_2_O (0.50 %); + (Fe+Zn), Foliar application of FeSO_4_.7H_2_O (0.25 %) + ZnSO_4_.7H_2_O (0.50 %)

**Supplementary table 2** Percentage increase using foliar application of FeSO_4_.7H_2_O, ZnSO_4_.7H_2_O (0.50 %) and their combination on GFeC, GZnC and GPC over control of ten wheat cultivars grown at Karnal during 2017-20

| Cultivars | GFeC (mg/kg) | | | GZnC (mg/kg) | | | GPC (%) | | |
| --- | --- | --- | --- | --- | --- | --- | --- | --- | --- |
|  | + Fe | + Zn | + (Fe+Zn) | + Fe | + Zn | + (Fe+Zn) | + Fe | + Zn | + (Fe+Zn) |
| DBW 173 | 8.1 | 7.3 | 5.2 | 4.9 | 42.1 | 56.9 | 4.2 | 3.3 | 1.6 |
| DBW 88 | 9.8 | 3.7 | 4.2 | 13.4 | 33.0 | 43.3 | 7.7 | 6.7 | 4.1 |
| DBW 90 | 6.8 | 9.2 | 11.0 | 2.5 | 36.0 | 42.6 | 7.5 | 5.4 | 2.6 |
| DPW 621-50 | 3.7 | 3.1 | 6.6 | 4.6 | 40.7 | 43.2 | 7.5 | 3.6 | 2.8 |
| HD 2967 | 0.6 | 4.5 | 0.7 | 3.9 | 31.0 | 26.5 | 8.7 | 3.8 | 7.9 |
| HD 3086 | 2.4 | 3.5 | 4.0 | 5.3 | 35.0 | 36.4 | 9.0 | 4.8 | 4.3 |
| HPBW 01 | 5.3 | 8.0 | 8.6 | 5.0 | 53.0 | 42.4 | 10.2 | 10.2 | 1.4 |
| K 307 | 5.0 | 3.7 | 10.5 | 2.8 | 35.5 | 51.8 | 6.5 | 6.2 | 2.7 |
| WB 2 | 3.3 | 6.5 | 4.3 | 5.1 | 45.5 | 50.5 | 3.8 | 4.0 | 2.9 |
| WH 1105 | 5.9 | 11.6 | 3.9 | 4.3 | 43.8 | 44.9 | 3.0 | 0.8 | 2.6 |

GFeC, Grain iron concentration; GZnC, Grain zinc concentration; GPC, Grain protein content; + Fe, Foliar application of FeSO_4_.7H_2_O (0.25 %); +Zn, Foliar application of ZnSO_4_.7H_2_O (0.50 %); + (Fe+Zn), Foliar application of FeSO_4_.7H_2_O (0.25 %) + ZnSO_4_.7H_2_O (0.50 %)
